# Survival Trend in Individuals With De Novo Metastatic Prostate Cancer After the Introduction of Doublet Therapy

**DOI:** 10.1001/jamanetworkopen.2023.36604

**Published:** 2023-10-02

**Authors:** Christian Corsini, Hans Garmo, Andri Wilberg Orrason, Rolf Gedeborg, Pär Stattin, Marcus Westerberg

**Affiliations:** 1 Department of Surgical Sciences, Uppsala University, Uppsala, Sweden; 2 Division of Experimental Oncology/Unit of Urology, URI Institution, IRCCS San Raffaele Hospital, Milan, Italy; 3 Medical Products Agency, Stockholm, Sweden

## Abstract

**Importance:**

Recently, life-prolonging treatments for patients with advanced prostate cancer have been introduced in clinical practice.

**Objective:**

To investigate if the introduction of doublet therapy is associated with changes in survival on a population-basis.

**Design, Setting, and Participants:**

This nationwide population-based cohort study used data from the Prostate Cancer data Base Sweden from 2008 to 2020. Men registered with de novo metastatic castration-sensitive prostate cancer (mCSPC) were included.

**Exposure:**

The proportion of men with mCSPC who received doublet therapy, ie, androgen deprivation therapy plus androgen receptor pathway inhibitor drugs or chemotherapy was assessed.

**Main Outcomes and Measures:**

Standardized overall survival, taking age, comorbidity, and cancer characteristics into consideration, was estimated by use of a parametric survival model.

**Results:**

A total of 11 382 men were included in this study (median [IQR] age, 74.0 [68-81] years). There was a shift toward less advanced prostate cancer during the study period with a decrease in median (IQR) prostate-specific antigen at diagnosis in men with mCSPC from 145 (39-571) ng/mL to 107 (27-426) ng/mL. Upfront treatment with doublet therapy in these men simultaneously increased from 1% (7 of 991) in 2016 to 44% (402 of 922) in 2020. The adjusted 5-year overall survival increased from 26% (95% CI, 25%-28%) from 2008 to 2012 to 35% (95% CI, 31%-40%) from 2017 to 2020. During the first 5 years after diagnosis, there was an increase in mean survival of 6 months, from 2.7 (95% CI, 2.6-2.8) years from 2008 to 2012 to 3.2 (95% CI, 3.1-3.1) years from 2017 to 2020.

**Conclusions and Relevance:**

In parallel with improvements in treatment of advanced prostate cancer, a clinically meaningful increase in mean survival was observed in men with de novo mCSPC in Sweden between 2008 and 2020 in this study.

## Key Points

**Question:**

Has the introduction of doublet therapy in individuals with de novo metastatic castration-sensitive prostate cancer been associated with changes in survival on a population basis in Sweden?

**Findings:**

In this nationwide cohort study, upfront treatment with doublet therapy among 11 382 individuals between 2008 and 2020 with de novo metastatic castration-sensitive prostate cancer increased from 1% in 2016 to 44% in 2020. Mean survival in individuals with de novo metastatic castration-sensitive prostate cancer increased 6 months during the first 5 years of follow-up.

**Meaning:**

In parallel with improvements in treatment of advanced prostate cancer, a clinically meaningful increase in mean survival was observed in this study.

## Teaser

This cohort study investigates if the introduction of doublet therapy is associated with changes in survival in individuals with de novo metastatic castration-sensitive prostate cancer.

## Introduction

In randomized clinical trials (RCT), the addition of docetaxel or an androgen receptor pathway inhibitor (ARPi) drugs to standard androgen deprivation therapy (ADT), ie, doublet therapy, increased survival in individuals with de novo metastatic castration-sensitive prostate cancer (mCSPC), ie, metastatic disease at diagnosis.^1,2,3,4,5,6,7,8,9^ The improvement was most pronounced in patients with high-volume disease.^1,10^ In the STAMPEDE trial, abiraterone acetate with prednisone improved 5-year overall survival to 60% compared with 41% in the group treated with ADT alone.^6^ Similarly, in the LATITUDE trial, median overall survival was 53.3 months in men with de novo metastatic high-risk cancer treated with doublet therapy with abiraterone acetate, compared with 36.5 months in the placebo group.^7^ Novel ARPis such as enzalutamide and apalutamide, when combined with ADT, have also been demonstrated to significantly increase progression-free survival^11^ and overall survival.^8,9^

Consequently, guidelines from the European Association of Urology and the National Swedish Guidelines now recommend doublet therapy for men with mCSPC.^12,13^ As a result, use of doublet therapy has increased substantially in Sweden, and in 2021 approximately half of all individuals with de novo mCSPC received doublet therapy. At the same time, earlier detection of metastatic disease has led to lower tumor burden in patients with de novo mCSPC, as mirrored by lower prostate-specific antigen (PSA) levels at diagnosis.^14^ The aim of this study was to investigate if the increased use of doublet therapy in men with de novo mCSPC in Sweden has been accompanied by improvements in survival, taking other temporal changes into consideration.

## Methods

The National Prostate Cancer Register (NPCR) of Sweden captures 98% of all incident prostate cancer cases compared with the Swedish Cancer Registry to which reporting is mandated by law.^15^ In the Prostate Cancer data Base Sweden (PCBaSe), NPCR has been enriched with data from other registers including the Patient Registry, the Cause of Death Registry, and the Prescribed Drug Registry, by use of the Swedish person identity number as previously described in detail.^16^ The Swedish Research Ethics Authority approved the study. The requirement for informed consent was waived by this authority. The study adheres to the Strengthening the Reporting of Observational Studies in Epidemiology (STROBE) reporting guideline.

### Study Population

The study population consisted of men registered from 2008 to 2020 in NPCR with de novo mCSPC defined by the presence of skeletal or visceral metastases on radionuclide bone scan, computed tomography, positron emission tomography (PET)/computed tomography, magnetic resonance imaging, or radiograph imaging.

### Exposure

Data on use of the ARPi drugs abiraterone (Anatomical Therapeutic Chemical code L02BX03), enzalutamide (L02BB04), and apalutamide (L02BB05) were based on filled prescriptions for these drugs in the Prescribed Drug Registry. Data on treatment with docetaxel were available in NPCR from March 2017. Upfront treatment was defined as treatment given within 6 months from diagnosis. The use of ARPis was further assessed at 6 to 24 months from date of diagnosis.

### Prostate Cancer Characteristics

Data were extracted from NPCR on PSA, mode of cancer detection (nonorganized screening or workup of men with low urinary tract symptoms, bone pain, or hematuria), Gleason score, clinical stage according to TNM (tumor, node, metastasis) classification, and primary treatment.

### Comorbidity

We estimated life expectancy at the time of diagnosis by use of age and 2 measures of comorbidity, a drug comorbidity index (DCI) and a newly created multidimensional comorbidity index (MDCI).^17^ MDCI was based on hospital discharge diagnoses registered in the National Patient Registry during 10 years prior to date of prostate cancer diagnosis, while the DCI was based on filled prescriptions in the Prescribed Drug Registry during the year prior to diagnosis. We optimized a previously described method to calculate the life expectancy by using the MDCI instead of Charlson Comorbidity Index in the model.^18^

### Outcomes

Outcomes were overall survival and cause-specific survival according to date and cause of death as registered in the Cause of Death Registry. Follow-up started at date of diagnosis and ended on December 31, 2022, or at date of death, whichever event came first.

### Statistical Analysis

Survival was estimated annually and in 3 calendar periods corresponding to the gradual uptake of doublet therapy, ie, 2008 to 2012, 2013 to 2016, and 2017 to 2020. All analyses were stratified according to age at diagnosis (<60, 60-69, 70-79, and ≥80 years). Crude survival was described with Kaplan-Meier curves. Differences in survival due to heterogeneous treatment intensity over calendar periods were expected to become evident after 6 months from start of treatment so hazards were not expected to be proportional.^19^ We estimated standardized survival curves using a parametric gamma survival model allowing for nonproportional hazards, comparing the 3 study periods.^20^ We standardized according to the case mix of men diagnosed from 2017 to 2020 by adjusting for age, PSA, Gleason score, clinical T stage, mode of cancer detection, primary treatment, and comorbidity by use of DCI and MDCI. Adjusted annual survival was estimated similarly, standardized according to the case mix of men diagnosed in 2020, and the 10-year survival trend was estimated after 2022 for men diagnosed between 2013 and 2020. To estimate the magnitude of difference in survival between the 3 calendar periods and annually, we calculated the restricted mean survival at 5 and 10 years.^21,22^ Model fit was assessed by comparing the parametric survival curves with the corresponding Kaplan-Meier curves.

Missing data for PSA, T stage, Gleason score, primary treatment, and mode of detection was imputed (5 times) using multiple imputation.^23^ Confidence intervals were computed by use of bootstrapping (500 resamplings) followed by multiple imputation with the boot multiple imputation percentile method.^24^ Statistical analyses were performed with R version 3.5.3 (R Foundation).

## Results

### Baseline Characteristics

Age at diagnosis and burden of comorbidities of the 11 382 men diagnosed with de novo mCSPC remained essentially stable during the study from 2008 to 2020 (median [IQR] age, 74.0 [68-81] years) (Table). However, there was a shift toward less advanced prostate cancer, eg, the proportion of men with T4 tumors decreased slightly from 20% (694 of 3465) from 2008 to 2012 to 16% (344 of 3973) from 2017 to 2020, and the proportion of men with symptoms at diagnosis decreased from 83% (2870 of 3465) to 68% (2700 of 3973). The median (IQR) PSA at diagnosis decreased from 145 (39-571) ng/mL (to convert to micrograms per liter, multiply by 1) from 2008 to 2012 to 107 (27-426) ng/mL from 2017 to 2020. In men aged 75 to 79 years, median PSA decreased from 125 ng/mL to 58 ng/mL, and for men 80 years or older from 200 ng/mL to 116 ng/mL, whereas virtually no change was observed for men 74 years or younger (eFigure 1 in Supplement 1). Among men diagnosed with de novo metastatic disease, there were only minor changes in use of imaging techniques during the study period (eTable 1 in Supplement 1).

**Table.** Baseline Characteristics of Men Diagnosed With De Novo Metastatic Castration-Sensitive Prostate Cancer in 2008-2020 and Registered in the National Prostate Cancer Register of Sweden

Characteristic	Participants, No. (%)			
	2008-2012	2013-2016	2017-2020	2008-2020
No. of patients	3465	3944	3973	11 382
Age at diagnosis, y				
Median (IQR)	74.0 (67-81)	74.0 (68-81)	74.0 (69-80)	74.0 (68-81)
<65	607 (18)	572 (15)	495 (12)	1674 (15)
65-74	1202 (35)	1503 (38)	1504 (38)	4209 (37)
75-79	599 (17)	741 (19)	847 (21)	2187 (19)
>80	1057 (31)	1128 (29)	1127 (28)	3312 (29)
Life expectancy, y^a^				
Median (IQR)	10.8 (6.8-16.3)	11.2 (6.9-16.2)	10.8 (6.9-15.3)	10.9 (6.9-15.9)
≤5	489 (14)	562 (14)	587 (15)	1638 (14)
6-10	1073 (31)	1150 (29)	1203 (30)	3426 (30)
11-15	832 (24)	1031 (26)	1152 (29)	3015 (26)
>15	1071 (31)	1201 (30)	1031 (26)	3303 (29)
PSA at diagnosis, ng/mL				
Median (IQR)	145 (39-571)	110 (29-412)	107 (27-426)	119 (31-469)
<3	47 (1)	50 (1)	54 (1)	151 (1)
3-20	457 (13)	689 (17)	698 (18)	1844 (16)
20-49	475 (14)	589 (15)	668 (17)	1732 (15)
50-99	449 (13)	530 (13)	498 (13)	1477 (13)
100-199	496 (14)	536 (14)	552 (14)	1584 (14)
≥200	1495 (43)	1497 (38)	1462 (37)	4454 (39)
Missing	46 (2)	53 (2)	41 (1)	140 (1)
Clinical T stage				
T1	360 (10)	414 (10)	355 (9)	1129 (10)
T2	687 (20)	882 (22)	1002 (25)	2571 (23)
T3	1590 (46)	1844 (47)	1786 (45)	5220 (46)
T4	694 (20)	646 (16)	644 (16)	1984 (17)
Missing	134 (4)	158 (4)	186 (5)	478 (4)
Gleason score				
6	152 (4)	144 (4)	44 (1)	340 (3)
7 (3 + 4)	243 (7)	274 (7)	184 (5)	701 (6)
7 (4 + 3)	460 (13)	527 (13)	438 (11)	1425 (13)
8	892 (26)	807 (20)	717 (18)	2416 (21)
9	1142 (33)	1596 (40)	1906 (48)	4644 (41)
10	158 (5)	183 (5)	201 (5)	542 (5)
Missing	418 (12)	413 (11)	483 (12)	1314 (11)
Treatment^b^				
Conservative therapy	283 (8)	457 (11)	335 (9)	1075 (10)
Orchiectomy	536 (15)	295 (7)	128 (3)	959 (8)
Bicalutamide	173 (5)	258 (7)	215 (5)	646 (6)
GnRH agonist	2437 (70)	2876 (73)	3191 (80)	8504 (75)
Missing	36 (1)	58 (2)	104 (3)	198 (2)
Mode of detection				
Asymptomatic	520 (15)	863 (22)	1224 (31)	2607 (23)
Symptomatic	2870 (83)	3009 (76)	2700 (68)	8579 (75)
Missing	75 (2)	72 (2)	49 (1)	196 (2)

Abbreviations: GnRH, gonadotropin releasing hormone agonist; PSA, prostate-specific antigen; T, tumor stage.

SI conversion factor: To convert PSA to micrograms per liter, multiply by 1.

^a^ Life expectancy is calculated basing on age and comorbidity measures: multidimensional comorbidity index and drug comorbidity index.

^b^ Except docetaxel or androgen receptor pathways inhibitors.

### Temporal Changes in Treatment

**Figure 1.**
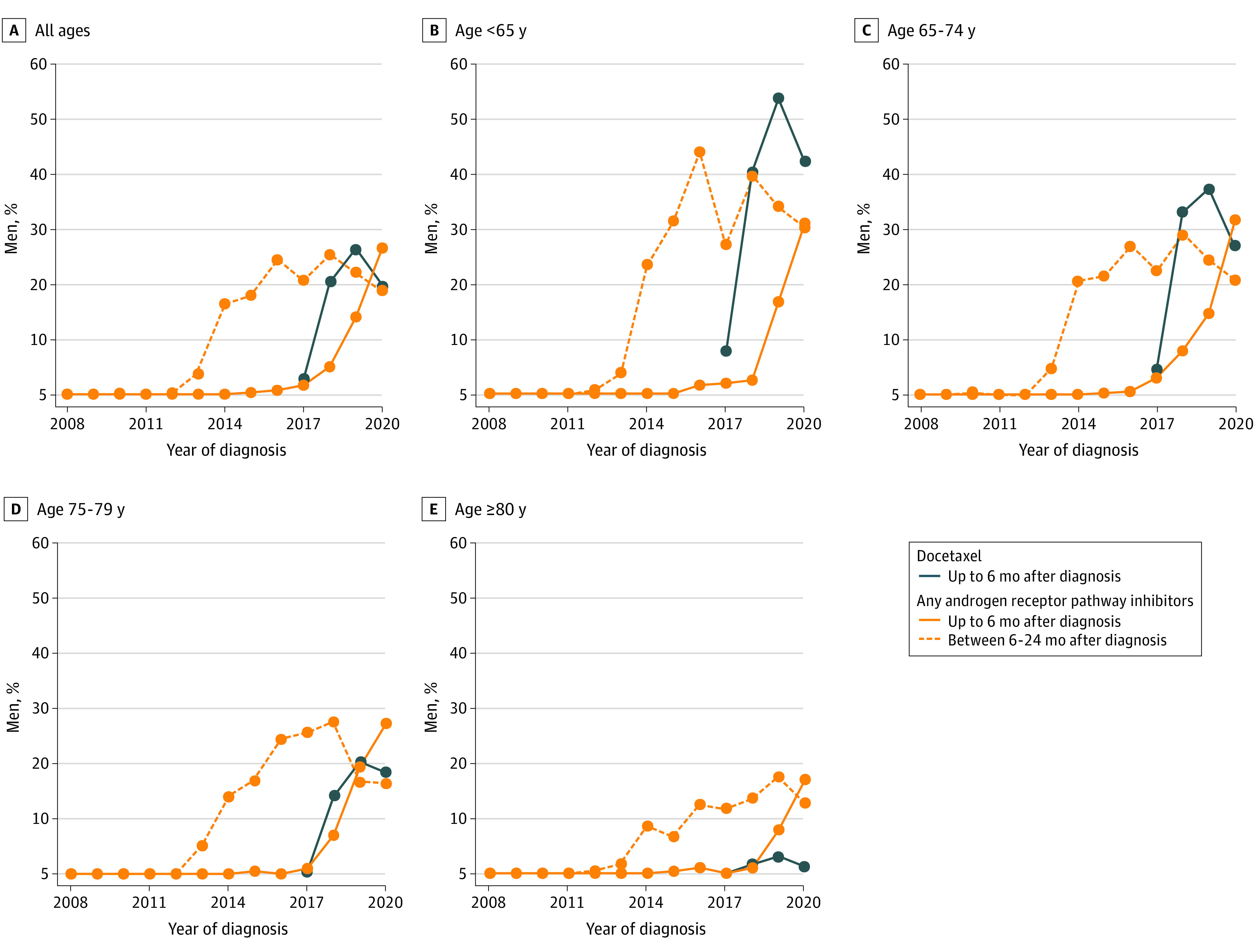
Use of Doublet Therapy in Men With De Novo Metastatic Castration-Sensitive Prostate Cancer, 2008-2020 Blue lines represent use of upfront docetaxel (data available from March 2017). Orange lines represent use of an androgen receptor pathway inhibitor starting within 6 months (solid line) or between 6 and 24 months after diagnosis (dashed line).

Upfront treatment with doublet therapy for de novo mCSPC increased from 1% (7 of 991) in 2016 to 44% (402 of 922) in 2020 (Figure 1). Upfront use of docetaxel increased from 3% (26 of 980) in 2017 to 20% (183 of 922) in 2020 and upfront use of ARPis increased from 1% (7 of 991) in 2016 to 27% (245 of 922) in 2020. Treatment with docetaxel and ARPis was more common in younger compared with older men. In 2017, 7% (8 of 107) of men younger than 65 years received docetaxel, while in 2020, the percentage was 42% (53 of 126). No man older than 80 years was treated with docetaxel in 2017 and 1% (3 of 258) was treated with docetaxel in 2020. Similarly, there was a stronger increase in use of ARPis among men younger than 65 years from 2% (2 of 132) in 2016 to 31% (39 of 126) in 2020 than for men older than 80 years, with corresponding percentages of 1% (3 of 296) and 17% (44 of 258). The use of ARPis 6 to 24 months after diagnosis was negligible prior to 2012 and increased to 19% (174 of 922) in 2020.

### Survival by Calendar Period

**Figure 2.**
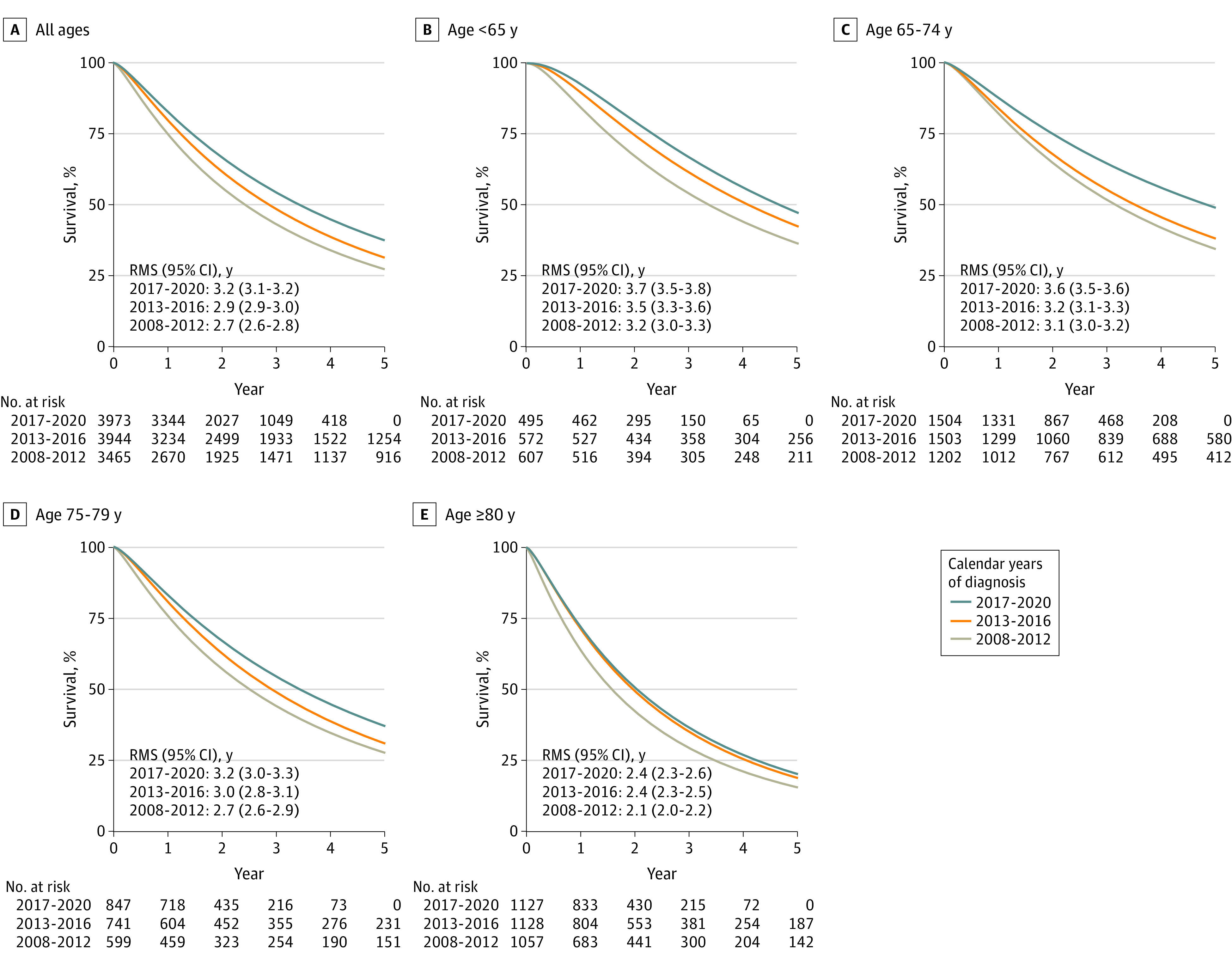
Standardized 5-Year Overall Survival for Men With De Novo Metastatic Castration-Sensitive Prostate Cancer Diagnosed in 2008-2020 Overall survival was estimated using a flexible parametric model standardized for baseline clinical characteristics according to last calendar period (2017-2020). Five-year restricted mean survival (RMS) was used to describe the increase in survival time. Survival estimates and 95% CI are also reported in eTable 1 in Supplement 1. Numbers at risk were extracted from the unadjusted analysis.

Standardized 5-year overall survival increased from 26% (95% CI, 25%-28%) from 2008 to 2012 to 35% (95% CI, 31%-40%) from 2017 to 2020. This corresponded to an increase of 6 months in mean survival 5 years after diagnosis, from 2.7 years (95% CI, 2.6-2.8 years) to 3.2 years (95% CI, 3.1-3.2 years) (Figure 2; eTable 2 in Supplement 1). In men older than 80 years, the increase was less pronounced, with an increase of 3.6 months in mean survival.

The parametric survival models fitted the observed survival data described by the Kaplan-Meier curves well (eFigure 2 and eTable 2 in Supplement 1). Cause-specific survival mirrored the overall survival, and the temporal trends were similar.

### Standardized 10-Year Survival Trend

**Figure 3.**
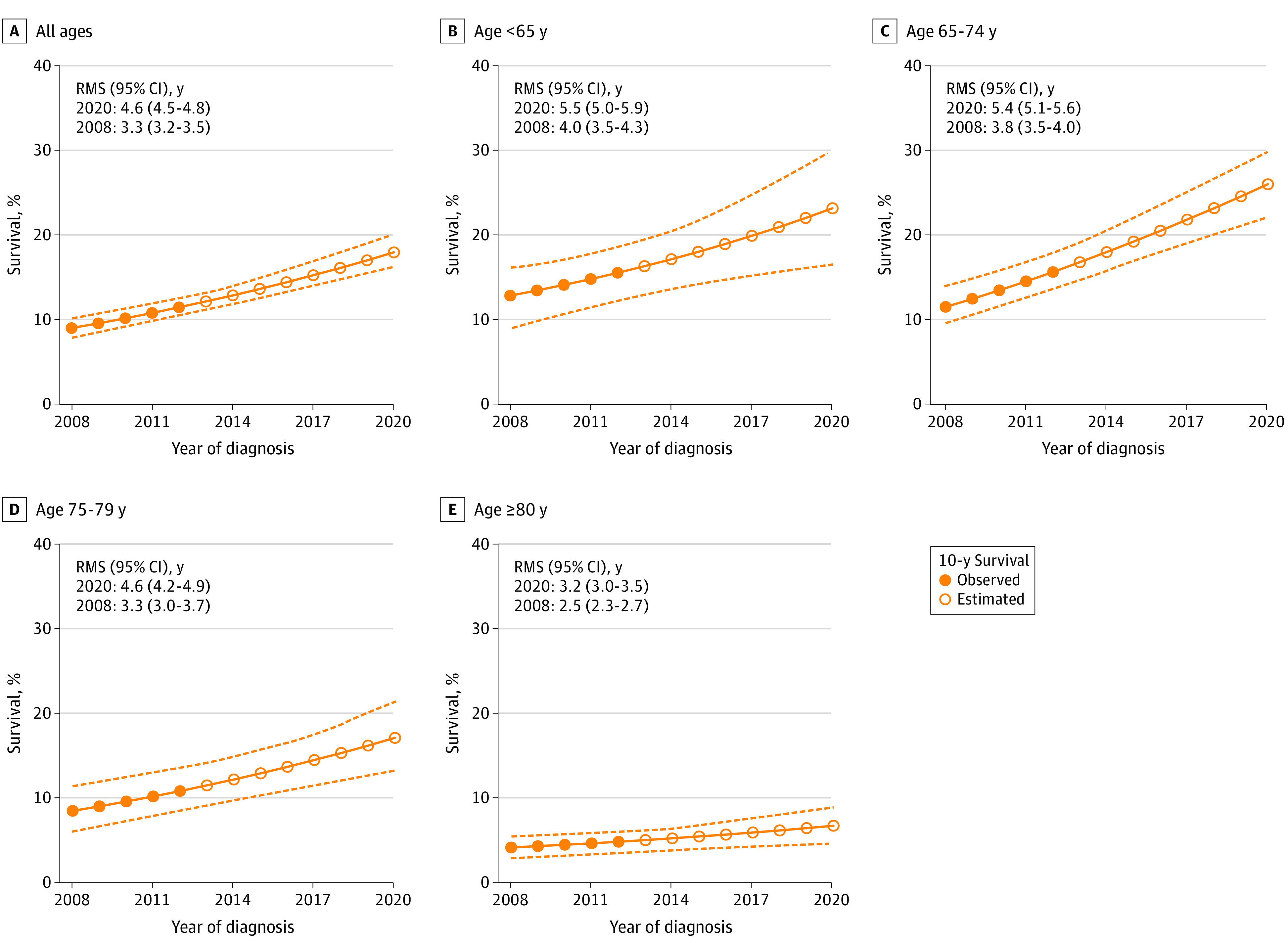
Standardized Trend for Observed and Estimated 10-Year Overall Survival in Men With De Novo Metastatic Castration-Sensitive Prostate Cancer Ten-year overall survival was estimated by use of a flexible parametric model standardized to baseline characteristics of men diagnosed in 2020. The 10-year restricted mean survival (RMS) was used to report the increase in survival time. Dashed lines represent lower and upper limits of the 95% CIs.

The 10-year overall survival increased from 9% (95% CI, 8%-10%) for men diagnosed in 2008 to 11% (95% CI, 11%-13%) for men diagnosed in 2012, with a further estimated increase to 18% (95% CI, 16%-20%) in 2020 (Figure 3). For the whole study group, an increase was estimated in mean survival at 10 years of follow-up from 3.3 (95% CI, 3.2-3.5) years for men diagnosed in 2008 to 4.6 (95% CI, 4.5-4.8) years for men diagnosed in 2020. The estimated survival increase was lower in men older than 80 years.

## Discussion

In this nationwide population-based study in Sweden, the addition of ARPi or docetaxel to standard ADT increased substantially between 2017 and 2020. In 2020, approximately 50% of patients with de novo mCSPC received doublet therapy. Between 2008 and 2020, mean survival increased with 6 months after 5 years of follow-up in all individuals with de novo mCSPC, taking changes in age, comorbidity, and cancer characteristics into account, supporting that doublet therapy is effective in clinical practice on a population basis.

### Standardization, Parametric Modeling, and Survival Estimation

Flexible parametric survival models were used to analyze survival, standardizing for changes in baseline characteristics during the study period and estimating long-term survival after 2022. The parametric gamma survival model allowed for nonproportional hazards that were expected since heterogeneous treatment intensity over calendar periods would affect survival only after around 6 months from start of treatment.^20^ Restricted mean survival is a useful measure to summarize changes in survival since it accounts for shape of the entire survival curve during follow-up in contrast to median survival that represents a snapshot of the survival at the time when 50% of men have died. The increase in restricted mean survival is likely a conservative estimate of the improvement in long-term survival since we expect the time period–specific survival curves to remain separated during most of the remaining follow-up. Nevertheless, our estimation of survival beyond the observed follow-up should be interpreted with caution particularly since it does not account for future changes in treatment that are likely to increase survival even further in individuals with mCSPC.

### Reasons for Improvement in Survival

In parallel to the introduction of doublet therapy, there continued to be a decrease in metastatic burden, mirrored by lower levels of PSA in individuals with mCSPC. This may partly explain the survival improvement in the last study period; however, the improvement remained after standardization for changes in cancer characteristics, including PSA levels. Furthermore, the biggest decrease in median PSA was observed among the oldest men for whom survival increased less than for the youngest men who received doublet therapy more often but whose median PSA did not decrease. There were minimal changes in the use of imaging during the study period, although there was a rise in prostate-specific membrane antigen PET use in the final year. Further studies are needed to determine the impact on survival of more sensitive imaging modalities, such as magnetic resonance imaging and prostate-specific membrane antigen PET.^25,26^

### Comparisons With RCTs

Unlike RCTs, observational studies may be biased from confounding, with fitter and younger men being more likely to receive more active treatment.^27,28,29,30^ To avoid this selection bias, we assessed survival in all men with de novo mCSPC, ie, including men who did not receive doublet therapy. In several RCTs, a substantially better survival has been observed for patients treated with ARPis or docetaxel compared with individuals receiving standard of care with ADT only, with the strongest effect observed when doublet therapy was used upfront.^2,7,8,9,31^ In the STAMPEDE trial, the 5-year overall survival was 60% in individuals undergoing ADT plus ARPi compared with 41% in individuals undergoing ADT only,^32^ whereas in our study, 5-year overall survival was 50% in men younger than 74 years in the latest calendar period, which seems reasonable given that about half of these men received doublet therapy. Men in our study population had comparable median PSA as men in the STAMPEDE trial (103 ng/mL vs 97 ng/mL), suggesting that the disease burden was quite similar.^5,33^

### Strengths and Limitations

This study has several strengths. The PCBaSe captures virtually all individuals diagnosed with prostate cancer in Sweden with registration of comprehensive data of cancer characteristics at diagnosis as well as primary treatment, complete capture of filled prescriptions, and complete follow-up of mortality. Comorbidity was assessed by use of 2 new indices based on comprehensive data in the Patient Registry and the Prescribed Drug Registry. The recency of the data is another strength of our study, with a last date of follow-up in December 2022.

There are also some limitations to our study. Although there were no substantial changes in the diagnostic workup, eg, imaging, unmeasured and unknown changes over calendar time may have affected survival. For example, information on the extent of bone metastases was only available from 2018, precluding us to analyze high- vs low-volume disease. Furthermore, there is no information on PSA levels during follow-up in NPCR or in any other nationwide register, so we could not assess progression-free survival. Upfront treatment with docetaxel has been captured in NPCR since March 2017; however, it was used before that date mostly among individuals with mCRPC. Use of docetaxel in patients with mCSPC was correctly registered in NPCR in 84% in an audit of 500 health care records. We did not have information on the use of docetaxel in individuals with mCRPC. However, the survival benefit of docetaxel in individuals with mCRPC is limited compared with upfront use in men with de novo mCSPC.^5,34^ Overall, 2% of patients were treated with both docetaxel and ARPi within the first 6 months. We do not know why both drugs were used nor do we know the date for start of docetaxel treatment. We speculate that intolerance to docetaxel made the clinician switch to an ARPi.

## Conclusions

A clinically meaningful increase in long-term survival was observed in men diagnosed with de novo mCSPC between 2008 and 2020 in Sweden. We argue that the main reason for this improvement was the increased upfront use of doublet therapy, combining ADT with docetaxel or an ARPi. Continued increase in use of doublet therapy and the introduction of triplet therapy (ADT plus docetaxel plus ARPis)^31^ will likely increase survival further in men with metastatic prostate cancer.

## Notes

Supplement 1. **eFigure 1.** Median and lower quartile level of PSA for men with de novo mCSPC in 2008-2020 **eFigure 2.** Unadjusted five-year overall survival for men with de novo mCSPC diagnosed in 2008-2020 **eTable 1. **Use of imaging techniques in men with de novo mCSPC in 2013-2020 in the National Prostate Cancer Register (NPCR) of Sweden **eTable 2.** Five-year crude survival in Kaplan-Meier analysis and standardized survival in parametric models

Supplement 2.
Data Sharing Statement
