# Supplementary material for: Survival Trend in Individuals With De Novo Metastatic Prostate Cancer After the Introduction of Doublet Therapy
Source: JAMA Netw Open. 2023 Oct 2;6(10):e2336604. doi: 10.1001/jamanetworkopen.2023.36604 (PMC10546238; doi:10.1001/jamanetworkopen.2023.36604)
Supplement: Supplement 1. — eFigure 1. Median and lower quartile level of PSA for men with de novo mCSPC in 2008-2020 eFigure 2. Unadjusted five-year overall survival for men with de novo mCSPC diagnosed in 2008-2020 eTable 1. Use of imaging techniques in men with de novo mCSPC in 2013-2020 in the National Prostate Cancer Register (NPCR) of Sweden eTable 2. Five-year crude survival in Kaplan-Meier analysis and standardized survival in parametric models [file jamanetwopen-e2336604-s001.pdf]

Supplemental Online Content

Corsini C, Garmo H, Orrason AW, Gedeborg R, Stattin P, Westerberg M. Survival trend in individuals with de novo metastatic prostate cancer after the introduction of doublet therapy. *JAMA Netw Open*. 2023;6(10):e2336604. doi:10.1001/jamanetworkopen.2023.36604

eFigure 1. Median and lower quartile level of PSA for men with de novo mCSPC in 2008-2020
eFigure 2. Unadjusted five-year overall survival for men with de novo mCSPC diagnosed in 2008-2020
eTable 1. Use of imaging techniques in men with de novo mCSPC in 2013-2020 in the National Prostate Cancer Register (NPCR) of Sweden
eTable 2. Five-year crude survival in Kaplan-Meier analysis and standardized survival in parametric models

This supplemental material has been provided by the authors to give readers additional information about their work.

© 2023 Corsini C et al. *JAMA Network Open.*

eTable 1: Use of imaging techniques in men with *de novo* mCSPC in 2013-2020 in the National Prostate Cancer Register (NPCR) of Sweden.

	2013 (N=949)	2014 (N=992)	2015 (N=1012)	2016 (N=991)	2017 (N=980)	2018 (N=1025)	2019 (N=1046)	2020 (N=922)
**Bone scan[1]**	645 (68%)	714 (72%)	739 (73%)	746 (75%)	756 (77%)	763 (74%)	798 (76%)	674 (73%)
**CT[1]**	112 (12%)	96 (10%)	81 (8%)	129 (13%)	124 (13%)	125 (12%)	123 (12%)	120 (13%)
**MR[1]**	47 (5%)	36 (4%)	39 (4%)	26 (3%)	30 (3%)	34 (3%)	24 (2%)	35 (4%)
**PET-CT[2]**	-	14 (1%)	25 (2%)	30 (3%)	37 (4%)	37 (4%)	46 (4%)	61 (7%)
**RX[3]**	-	-	8 (1%)	4 (0%)	1 (0%)	3 (0%)	2 (0%)	2 (0%)
**Unknown**	145 (15%)	132 (13%)	120 (12%)	56 (6%)	32 (3%)	63 (6%)	53 (5%)	30 (3%)

[1]Reported in NPCR since January 2013
[2]Reported in NPCR since June 2014
[3]Reported in NPCR since March 2015
CT= computed tomography; MRI=magnetic resonance imaging; PET-CT=positron emission tomography-CT; RX=X-ray imaging.

© 2023 Corsini C et al. *JAMA Network Open.*

eTable 2. Five-year crude survival in Kaplan-Meier analysis and standardized survival in parametric models.

			Crude survival in Kaplan-Meier analysis			Standardized survival in parametric models		
**Age**			**2008–2012**	**2013–2016**	**2017–2020**	**2008–2012**	**2013–2016**	**2017–2020**
All ages								
	OS	Estimate<br>95% CI	26<br>(25-28)	32<br>(30-33)	35<br>(31-40)	27<br>(26-28)	31<br>(30-32)	37<br>(36-39)
	CSS	Estimate<br>95% CI	35<br>(33-36)	42<br>(40-43)	48<br>(45-51)	35<br>(33-36)	39<br>(38-41)	45<br>(43-47)
<65 years								
	OS	Estimate<br>95% CI	35<br>(31-39)	45<br>(41-49)	50<br>(43-58)	36<br>(32-40)	42<br>(38-46)	47<br>(42-53)
	CSS	Estimate<br>95% CI	39<br>(35-43)	49<br>(45-54)	55<br>(48-64)	40<br>(35-44)	46<br>(42-50)	52<br>(46-59)
65–74 years								
	OS	Estimate<br>95% CI	34<br>(32-37)	39<br>(36-41)	51<br>(46-55)	34<br>(32-37)	38<br>(36-40)	49<br>(46-52)
	CSS	Estimate<br>95% CI	41<br>(39-45)	46<br>(44-49)	58<br>(54-63)	41<br>(38-43)	45<br>(42-47)	55<br>(52-59)
75–79 years								
	OS	Estimate<br>95% CI	25<br>(22-29)	31<br>(28-35)	34<br>(27-41)	27<br>(25-31)	31<br>(28-34)	37<br>(33-40)
	CSS	Estimate<br>95% CI	35<br>(31-39)	41<br>(37-45)	48<br>(42-54)	37<br>(33-41)	39<br>(36-43)	45<br>(41-50)
> 80 years								
	OS	Estimate<br>95% CI	13<br>(12-16)	17<br>(15-19)	11<br>(5-26)	15<br>(13-17)	19<br>(17-21)	20<br>(17-23)
	CSS	Estimate<br>95% CI	23<br>(20-26)	30<br>(27-34)	28<br>(21-36)	26<br>(23-29)	31<br>(28-33)	30<br>(27-34)

OS=overall survival; CSS=cause-specific survival; CI=confidence interval

© 2023 Corsini C et al. *JAMA Network Open.*

eFigure 1. Median and lower quartile level of PSA for men with *de novo* mCSPC in 2008-2020.

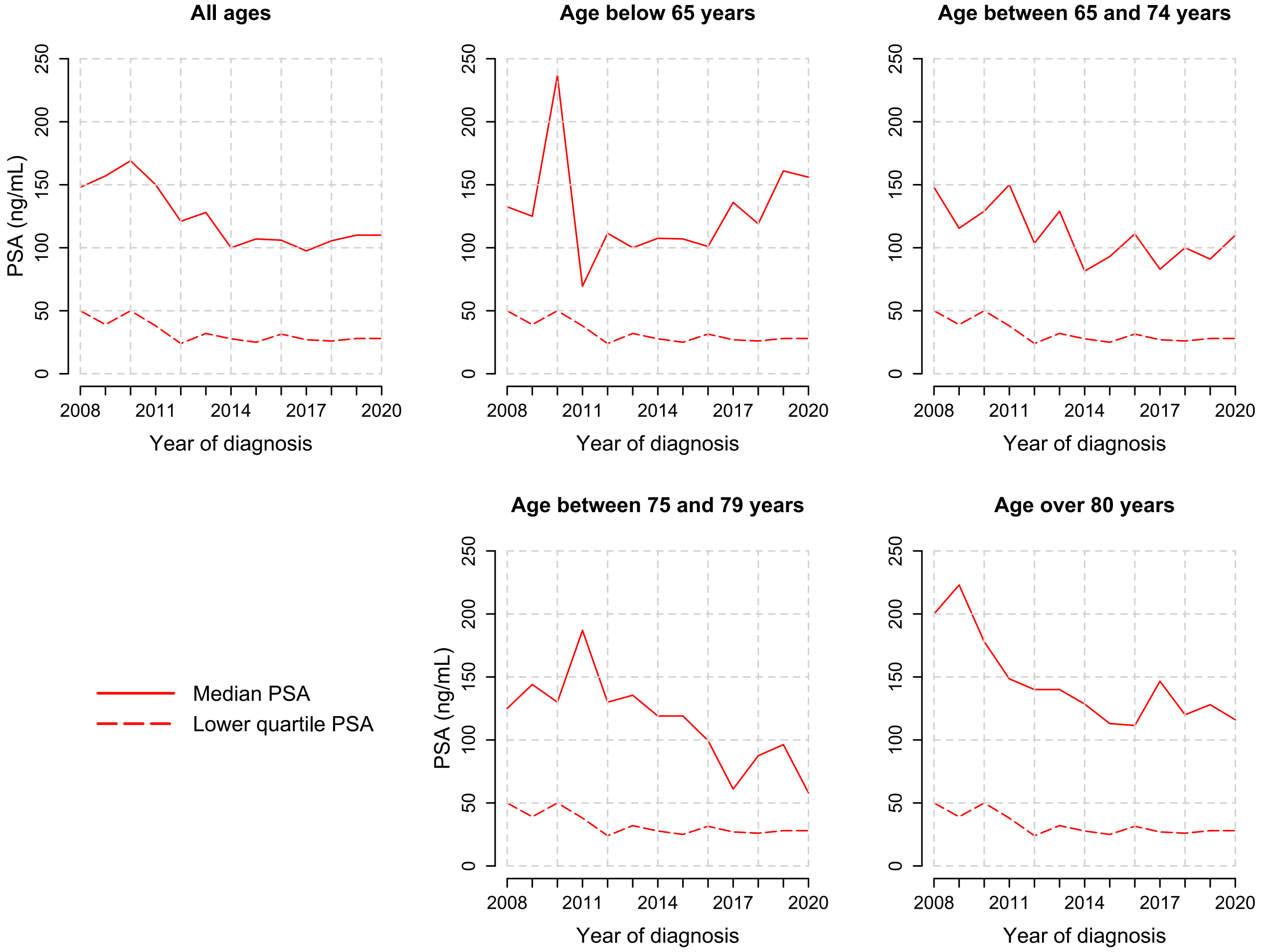

Legend: Solid lines represent median and dashed lines represent lower quartile. PSA=prostate specific antigen

© 2023 Corsini C et al. *JAMA Network Open.*

eFigure 2. Unadjusted five-year overall survival for men with *de novo* mCSPC diagnosed in 2008-2020.

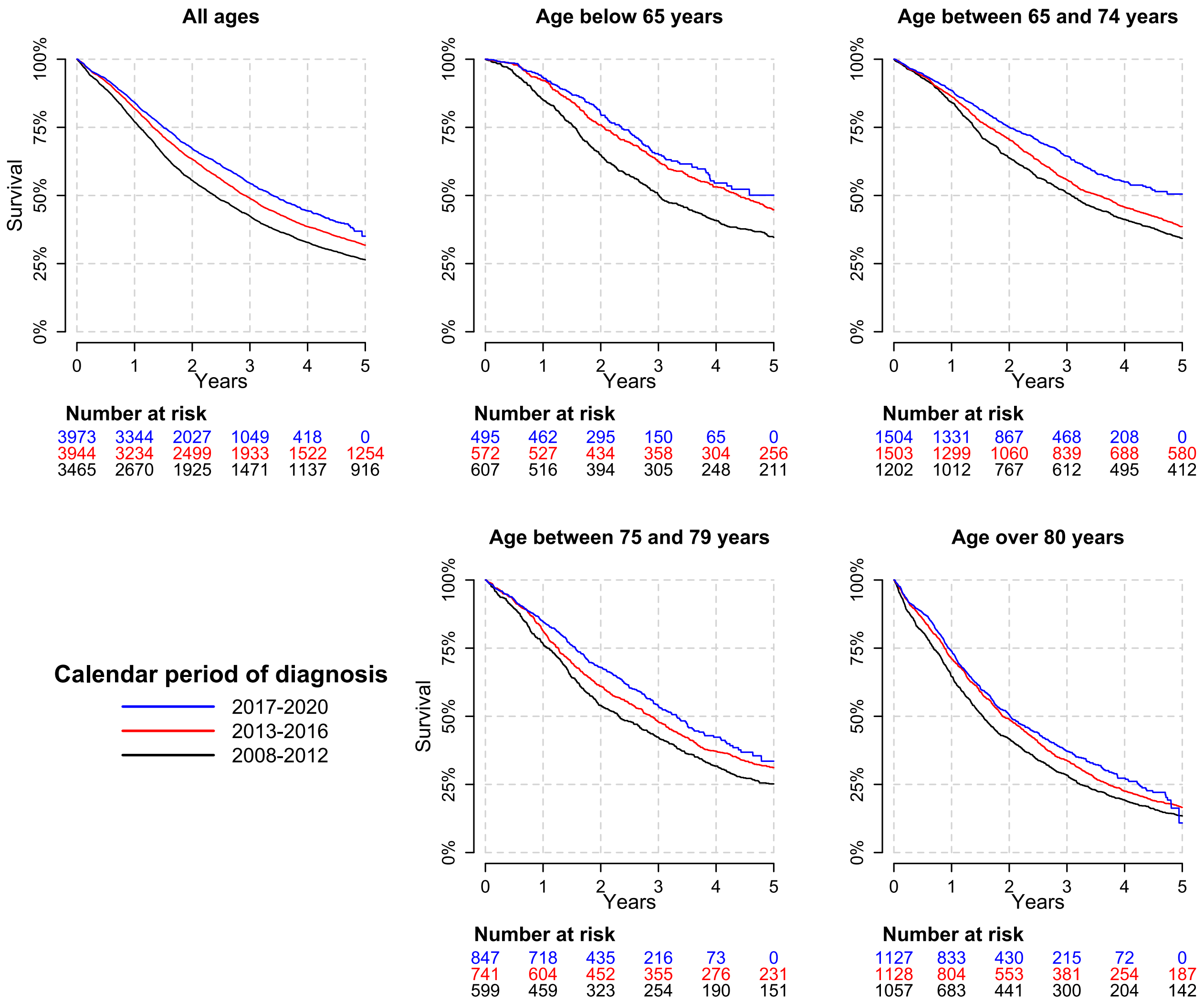

Legend: Overall survival calculated by use of Kaplan-Meier estimator. Survival estimates and 95% confidence intervals are reported in eTable 1.

© 2023 Corsini C et al. *JAMA Network Open.*
