# Supplementary material for: Survival Trend in Individuals With De Novo Metastatic Prostate Cancer After the Introduction of Doublet Therapy
Source: JAMA Netw Open. 2023 Oct 2;6(10):e2336604. doi: 10.1001/jamanetworkopen.2023.36604 (PMC10546238; doi:10.1001/jamanetworkopen.2023.36604)
Supplement: Supplement 2. — Data Sharing Statement [file jamanetwopen-e2336604-s002.pdf]

# Data Sharing Statement

Corsini. Survival Trend in Individuals With De Novo Metastatic Prostate Cancer After the Introduction of Doublet Therapy. *JAMA Netw Open*. Published October 02, 2023. doi:10.1001/jamanetworkopen.2023.36604

**Data**
**Data available:** No

**Additional Information**
**Explanation for why data not available:** Data used for the current study have been extracted from PCBaSe. Data can be made available on a remote server upon request to the PCBaSe reference group, contact par.stattin@.uu.se. The code used for the analyses can be provided on request by marcus.westerberg@uu.se.
